# The Paley ulnarization of the carpus with ulnar shortening osteotomy for treatment of radial club hand

**DOI:** 10.1051/sicotj/2016040

**Published:** 2017-01-24

**Authors:** Dror Paley

**Affiliations:** 1 Director, Paley Institute West Palm Beach Florida USA

**Keywords:** Radial club hand, Ulnarization, Radialization, Centralization, Congenital radius deficiency

## Abstract

Recurrent deformity from centralization and radialization led to the development in 1999 of a new technique by the author called ulnarization. This method is performed through a volar approach in a vascular and physeal sparing fashion. It biomechanically balances the muscle forces on the wrist by dorsally transferring the flexor carpi ulnaris (FCU) from a deforming to a corrective force. The previous problems of a prominent bump from the ulnar head and ulnar deviation instability were solved by acutely shortening the diaphysis and by temporarily fixing the station of the carpus to the ulnar head at the level of the scaphoid. This is the first report of this modified Paley ulnarization method, which the author considers a significant improvement over his original procedure.

## Introduction

Traditionally, radial club hand (RCH) treatment has been given to patients by centralization of the carpus on the end of the ulna [1–11]. Partial or complete recurrence of the radial clubhand deformity and growth arrest of the distal ulnar physis are common sequelae after centralization [1, 5, 11–15]. In 1985, Buck-Gramcko [11–13] described an alternative to centralization, which he termed *radialization*. He used a dorsal incision to dissect the hand free of the carpal bones and then translocated the hand to the ulnar side of the ulnar head. He also transferred and shortened the extensor and flexor carpi radialis (FCR) tendons respectively to the ulnar side to weaken the forces of radial deviation and strengthen the ulnar motors. The name *radialization*, which was coined in German, refers to conversion of the ulna into a radius rather than the direction of displacement of the carpus relative to the radius. The rate of partial growth arrest of the distal ulna (11% of cases) secondary to avascular necrosis of the growth plate and the rate of recurrence (7.5% of cases) were much lower than with centralization [13].

The biomechanical rationale of the radialization procedure was to create a fulcrum to the radial deviation forces of the muscles of the forearm while not impeding wrist motion. Furthermore, the idea was to balance the muscle forces on the radial and ulnar side of the wrist with the tendon transfers [11–13].

The Buck-Gramcko radialization is performed through a dorsal incision, without visualization of the neurovascular structures, excessive dissection of the ulna, and non-visualization of the volar radial vascular pedicle of the distal ulnar epiphysis and physis. Furthermore, the flexor and extensor carpi radialis tendons are usually absent and unavailable for transfer. The name *radialization* in English is confusing. It can be misunderstood to mean shifting the hand in a radial direction. Radialization moves the hand in an ulnar direction.

In 1999, the author developed a new procedure stimulated by the Buck-Gramcko radialization concept. Considering that centralization refers to centralizing the carpus on the ulna, Paley used his volar procedure *ulnarization* to describe the direction of movement of the carpus relative to the ulna and to distinguish it from radialization, which is performed differently [16–19]. The volar approach allows visualization and decompression of the neurovascular structures of the hand as well as identification and protection of the vascular pedicle of the ulnar epiphysis (caput ulna vessels). The other major difference is the dorsal transfer of the flexor carpi ulnaris (FCU) to augment the extensor moment on the wrist. Unlike the FCR, the FCU is always present and never hypoplastic.

**Figure 1.**
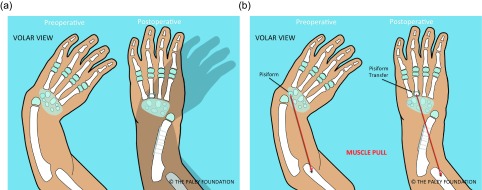
Pre- versus postoperative position of the hand following ulnarization with shortening (a). The muscle forces before surgery pull the hand into more radial deviation while after surgery there is a balance of forces with the ulnar head acting as a bony block to radial deviation (b).

The Paley ulnarization procedure has also undergone a significant evolution. Originally, the fixation was done with the Ilizarov circular external fixation apparatus. The head of the ulna was allowed to telescope distally as much as was needed to avoid excessive soft-tissue tension. This allowed complete angular correction according to the length of the tight soft tissues. After surgery, gradual distraction of the hand was carried out to transport the carpus distally, to articulate at the level of the scaphoid. This is the desired *station* for the new ulno-carpal articulation. It was then held in that position with the external fixator for another six weeks. Despite this, gradual proximal migration of the wrist would occur relative to the head of the ulna. This contributed to excessive ulnar deviation. In recent years, the author modified the original ulnarization technique to be able to pin the carpus adjacent to the ulnar head at *station* at the time of the index procedure (this idea was partially stimulated by discussions with Dr. David Feldman). This involved acutely shortening of the ulnar diaphysis with internal fixation (Figure 1).

## Methods

### New ulnarization surgical technique (Figures 2–24)

**Figure 2.**
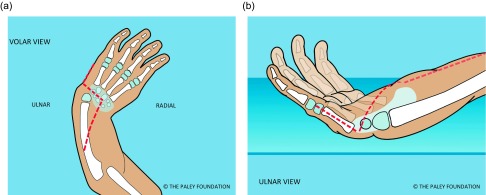
Make a Z-shaped incision. Shown from the volar aspect (a) and from the ulnar side (b).

Step 1: Under tourniquet control make a Z-shaped incision with the middle line of the Z, transverse across the wrist flexor crease, the proximal limb longitudinal from the midpoint of the wrist crease extending proximally and the distal limb along the ulnar border of the hand (Figure 2).

**Figure 3.**
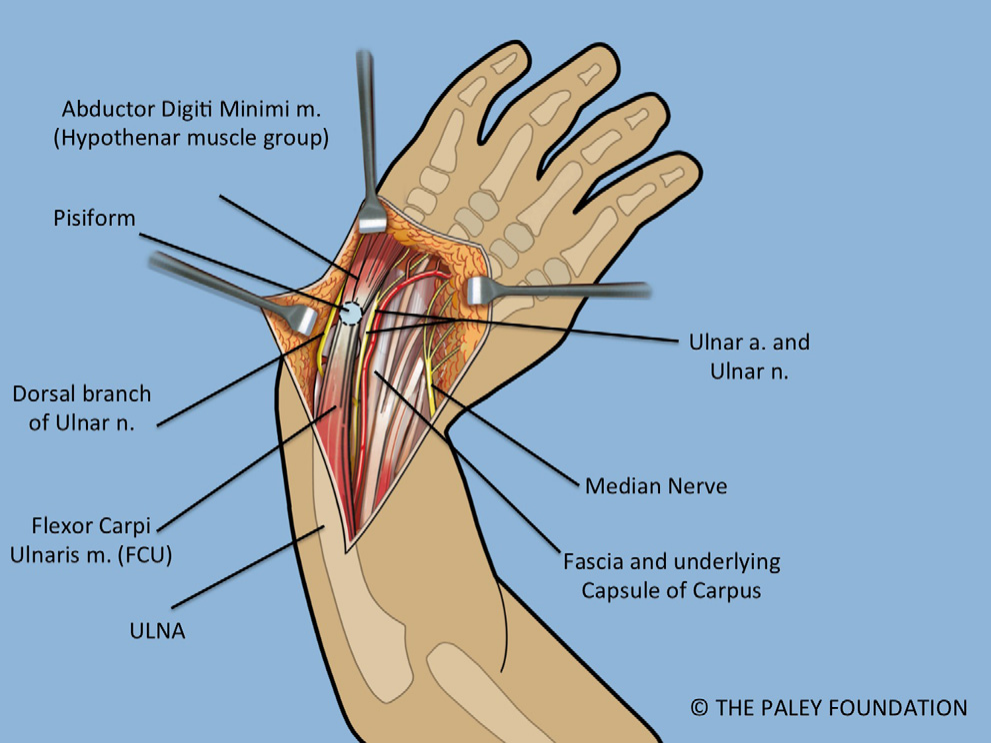
Volar forearm and wrist exposure after volar fasciotomy, showing the ulnar and median nerves, and the flexor tendons. Note the dorsal cutaneous branch of the ulnar nerve passes dorsal to the FCU.

Step 2: Elevate a full thickness volar ulnar proximal flap (Figure 3).

Step 3: Identify and expose the ulnar neurovascular bundle (Figure 3).

**Figure 4.**
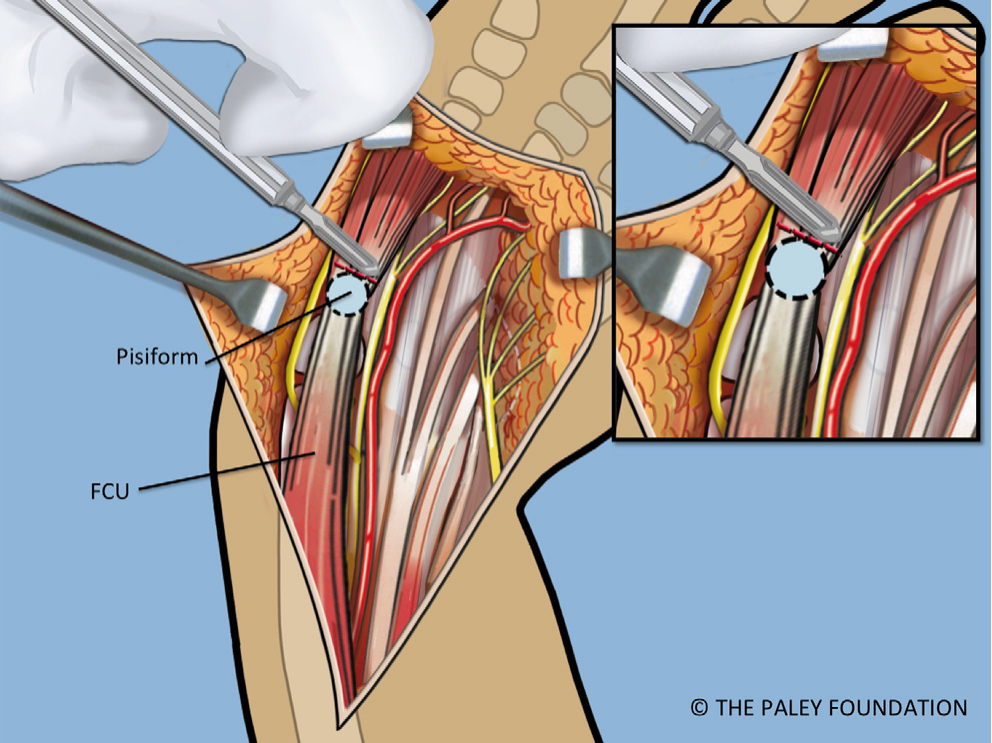
The abductor digiti minimi is released off of the pisiform bone. The pisiform bone is separated from the triquetrum.

Step 4: Follow the ulnar nerve distally and decompress it into the palm (Figure 4).

**Figure 5.**
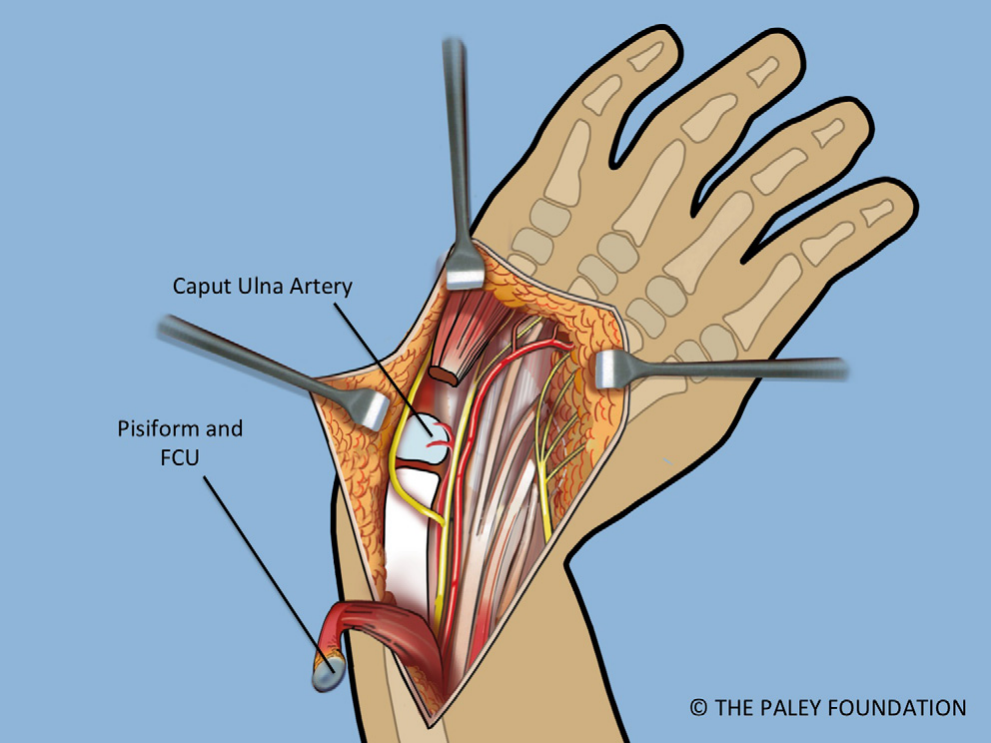
The FCU is reflected proximally. The circulation to the ulnar epiphysis comes from volar-radial. The ulnar and median nerves are decompressed into the hand.

Step 5: Expose the pisiform bone and dissect it free of the abductor and flexor muscles on its distal aspect. Release the capsule circumferentially between the pisiform and the triquetrum leaving only the FCU (flexor carpi ulnaris) tendon attachment proximally (Figures 4 and 5).

Step 6: The FCU tendon and muscle belly are dissected free of the ulna and reflected proximally with the attached pisiform bone. Care should be taken to identify the proximal neurovascular leash into the FCU muscle belly and to maintain its integrity. When dissecting the FCU tendon, the dorsal branch of the ulnar nerve must be identified and protected as it passes dorsal to the FCU (Figure 5).

Step 7: Identify the median nerve on the radial side of the forearm (Figures 4 and 5).

Step 8: Decompress the median nerve into the palm of the hand by cutting the flexor retinaculum distally (Figure 5).

**Figure 6.**
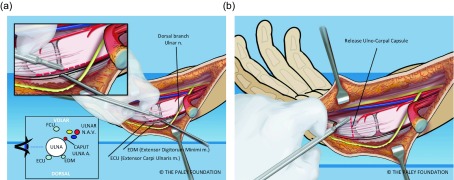
The ulna is dissected free of the ECU and EDM on its ulno-dorsal aspect (a). The ulnar collateral ligament and capsule are cut (b). The caput ulnar vessels lie on the volar radial aspect of the ulna.

Step 9: Expose the extensor carpi ulnaris (ECU) on the dorso-ulnar side of the ulna. Dissect it free proximally and distally to the base of the fifth metacarpal (Figure 6).

Step 10: Expose the extensor digiti minimi (EDM) on the dorso-radial aspect of the ulnar head. Free this tendon from the ulna. This tendon has a very small diameter and is very easy to injure during the dissection (Figure 6).

**Figure 7.**
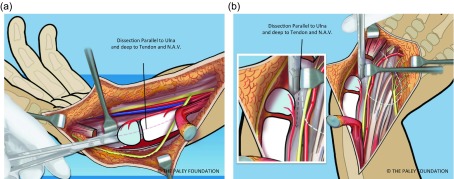
The capsule is released along the interval between the ulna and the carpus. The line of dissection is from distal to proximal, in line with the shaft of the ulna. Care must be taken to stay deep to the volar extra-articular soft tissues (a, b).

Step 11: Release the ulno-carpal capsule. The direction of dissection is not ulnar to radial but rather from distal to proximal parallel to the shaft of the ulna starting between the ulna and the carpus distally and working proximally. This will avoid cutting across the lunate. The location of the triquetrum serves as a landmark since its volar surface is exposed after removing the pisiform from the carpus (Figure 7).

**Figure 8.**
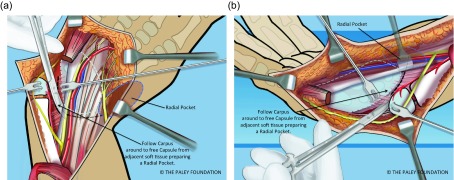
The capsular tissues are dissected by following around the proximal edge of the carpus towards the radial side (a, b).

Step 12: Continue this capsulotomy radially along the proximal border of the carpus. Avoid any dissection of the volar soft tissues connecting to the ulna. The vascular pedicle of the ulnar head is in this tissue mass. The ulna can be dissected free of soft tissues dorsally and on its ulnar side. The volar-radial soft tissues should be preserved. To cut the capsular connections between the ulna and carpus elevate and protect these tissues while cutting the capsule under direct vision (Figure 8).

**Figure 9.**
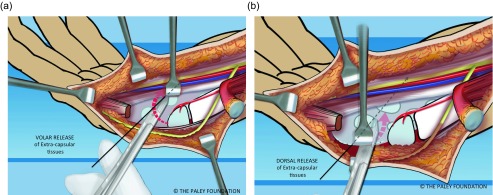
The volar (a) and dorsal (b) extra-capsular soft tissues are separated from the carpal bones by dissection.

Step 13: Dissect the volar and dorsal soft tissues off of the carpus. This dissection is quite extensive and may appear to almost devascularize the cartilage of the carpus. If the plane of dissection is slightly off of the plane of the microvasculature the carpal circulation survives. This part of the dissection separates the tendons on both sides of the carpus from the surface of the carpus. This makes the carpus mobile enough to allow its ulnarization relative to the ulnar head (Figure 9).

**Figure 10.**
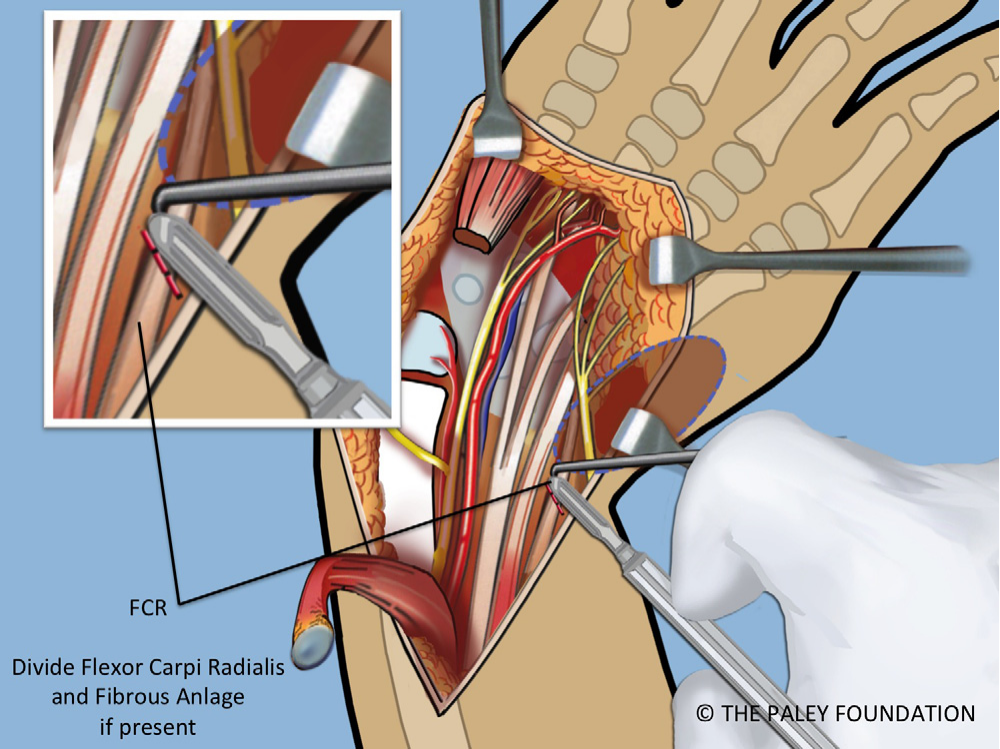
If the FCR is present it should be released. If a radial fibrous anlage is present it too should be released from the carpus.

**Figure 11.**
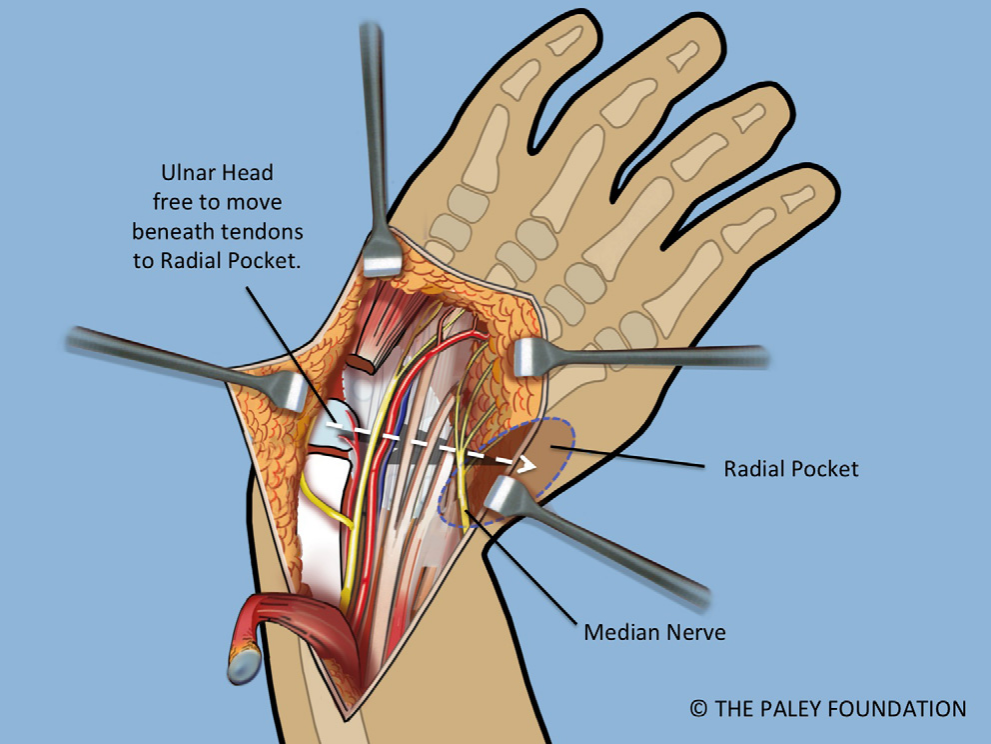
A space is made radial to the carpus, called the radial pocket.

Step 14: Dissect down to the radial aspect of the carpus and create a *radial pocket*, radial to the carpus. If the flexor carpi radialis tendon or an *anlage* of the radius is present, release it from the carpus (Figures 10 and 11).

**Figure 12.**
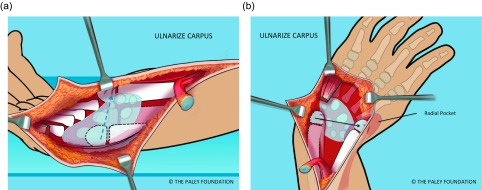
The wrist is ulnarized allowing the ulnar head to pass into the radial pocket (a, b).

Step 15: Ulnarize the carpus by moving it to the ulnar side of the ulnar head. The ulnar head passes under the volar tendons or deep to the dorsal tendons. There should be enough room to shift it into the radial pocket either volarly or ulnarly. Once in the radial pocket the hand is straight and the wrist seems stable (Figure 12).

**Figure 13.**
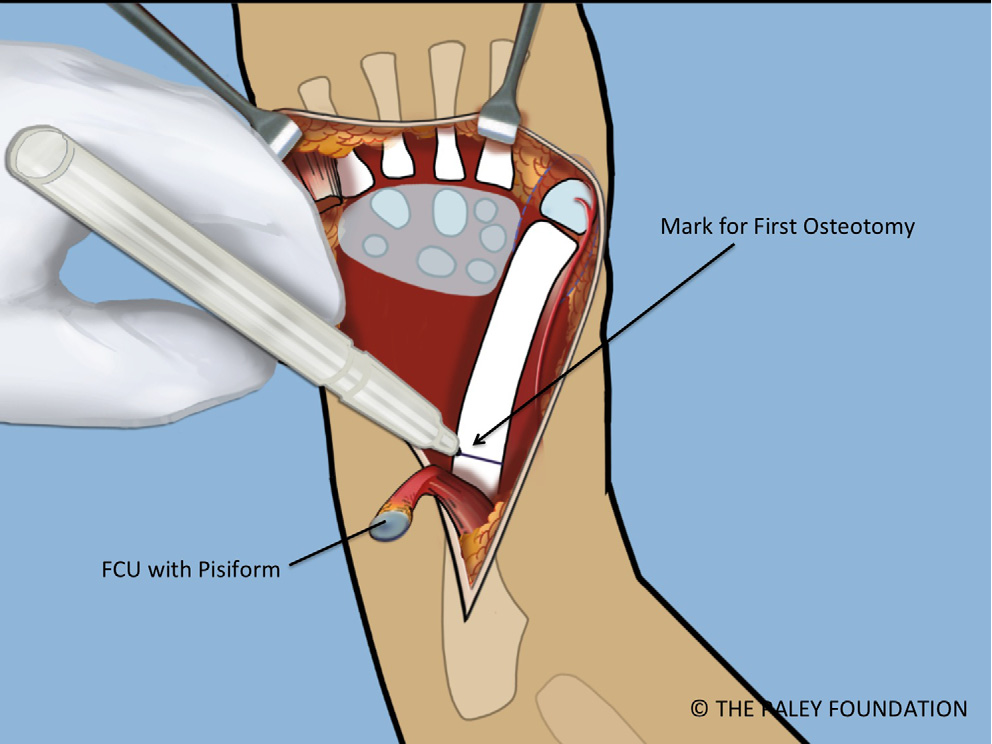
The ulnar diaphysis is exposed by elevating the FCU proximally. The level of the planned osteotomy is marked.

**Figure 14.**
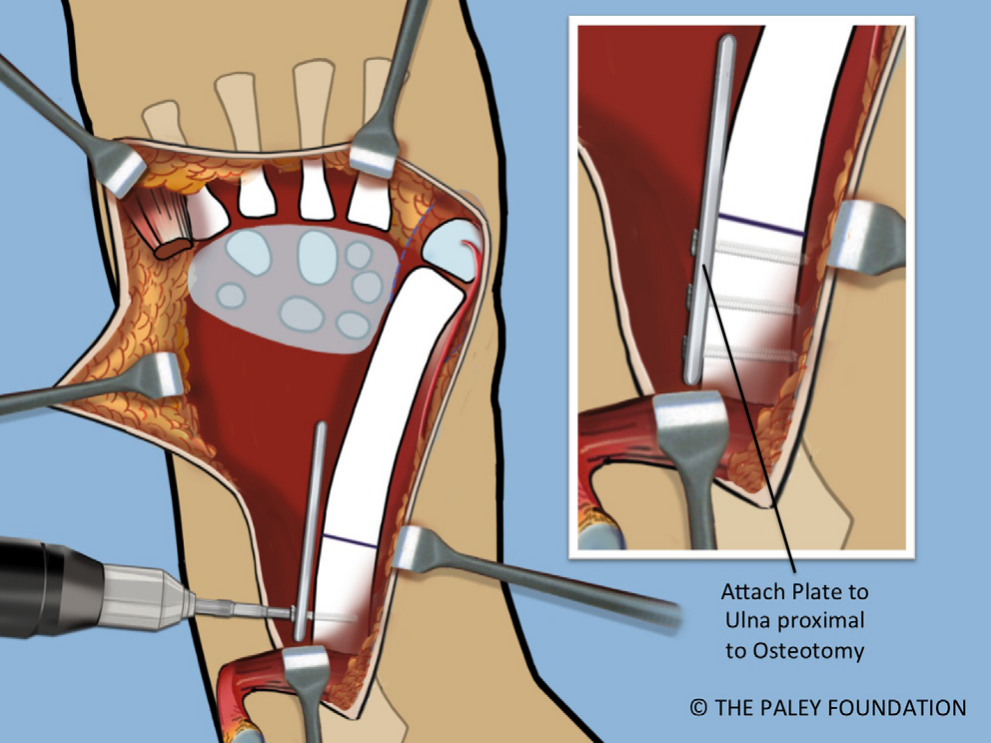
A small plate is affixed to the proximal ulna with two or three screws.

Step 16: Expose the shaft of the ulna by elevating the FCU. Take care not to damage the volarly based neurovascular bundle of this muscle. Mark the level of the planned osteotomy. Affix a small plate onto the ulna with two or three screws (Figures 13 and 14).

**Figure 15.**
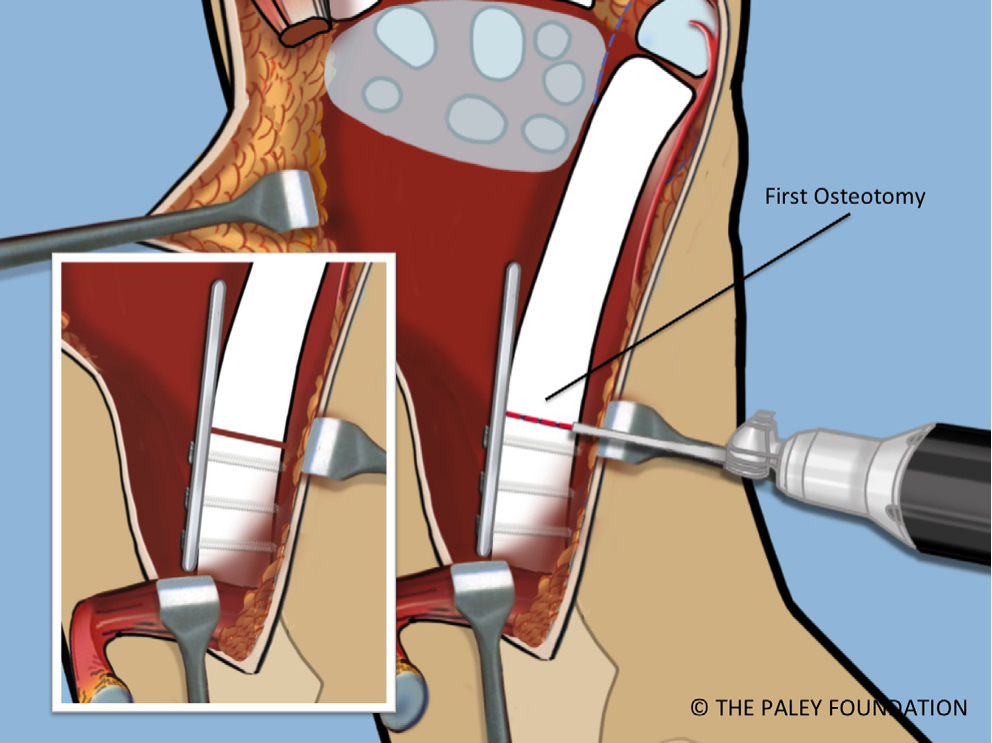
The proximal ulnar osteotomy is made using a saw.

Step 17: Cut the ulna in its mid-diaphysis with a saw (Figure 15).

**Figure 16.**
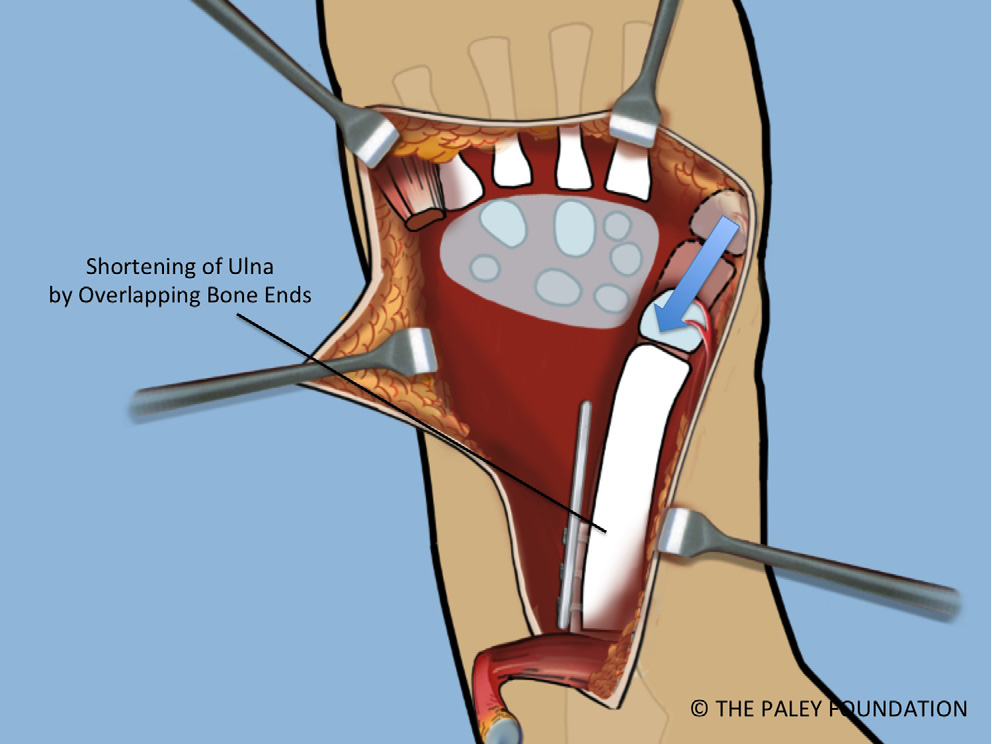
The distal ulna is stripped of some of its periosteum to allow the ulna to shorten and overlap the proximal end. Shortening of the ulna allows it to migrate proximally relative to the carpus. The ulnar head should sit opposite the scaphoid. This is referred to at *station.*

Step 18: Shorten the ulna and overlap the bone ends. Place the head of the ulna at the level desired relative to the carpus (scaphoid adjacent to ulnar head). This level is called at *station* (Figure 16).

**Figure 17.**
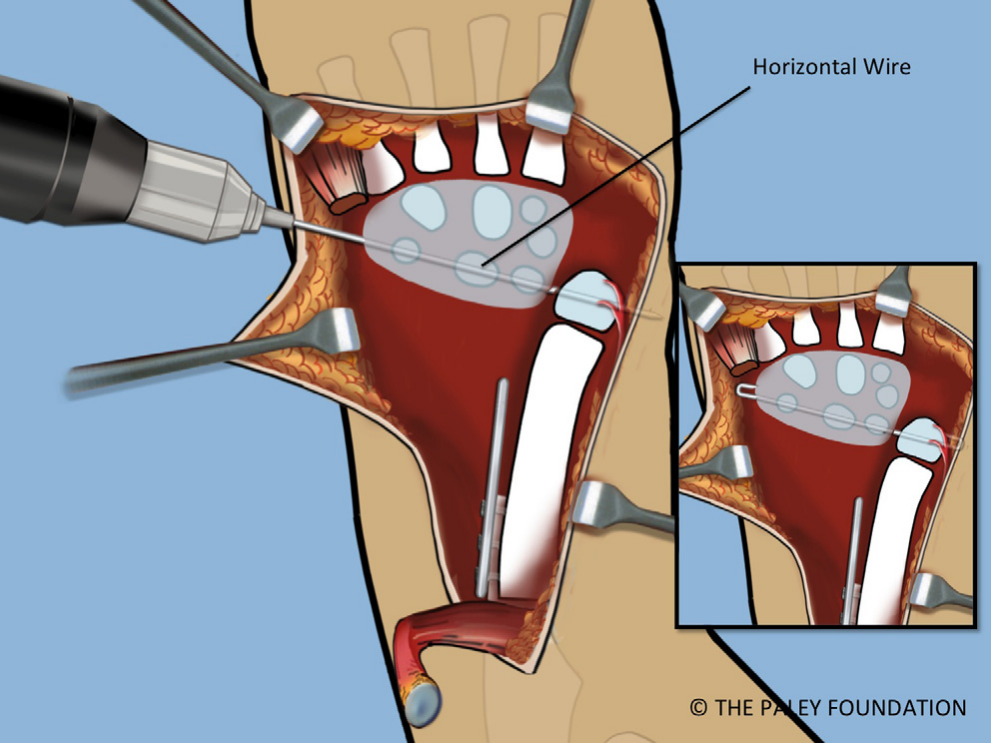
The carpus is pinned to the head of the ulna at station with a transverse 1.5 mm wire starting on the carpal side.

**Figure 18.**
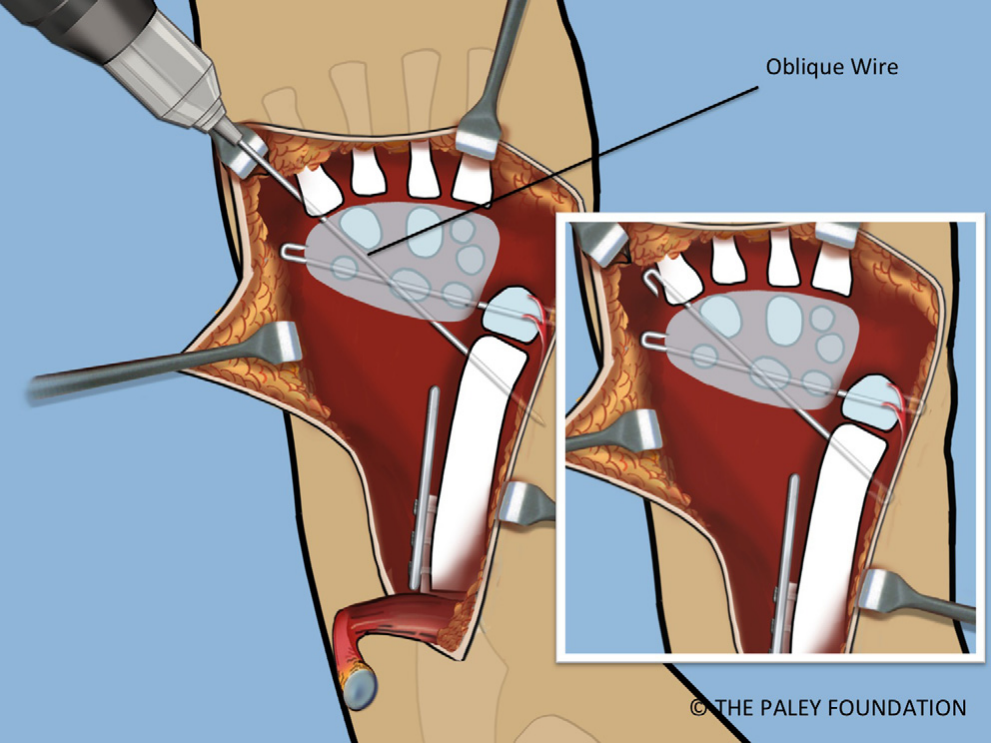
A second retrograde oblique wire is inserted from the fifth metatarsal base to the distal ulnar metaphysis. These wires should be cut and curled 180°.

Step 19: Pin the carpus to the head of the ulna with a 1.5 mm transverse wire. Insert a second wire obliquely from the base of the 5th metacarpal to the distal ulnar metaphysis. The hand should be neutral to slightly dorsiflexed. The ends of the wires should be curled over, cut and buried (Figures 17 and 18).

**Figure 19.**
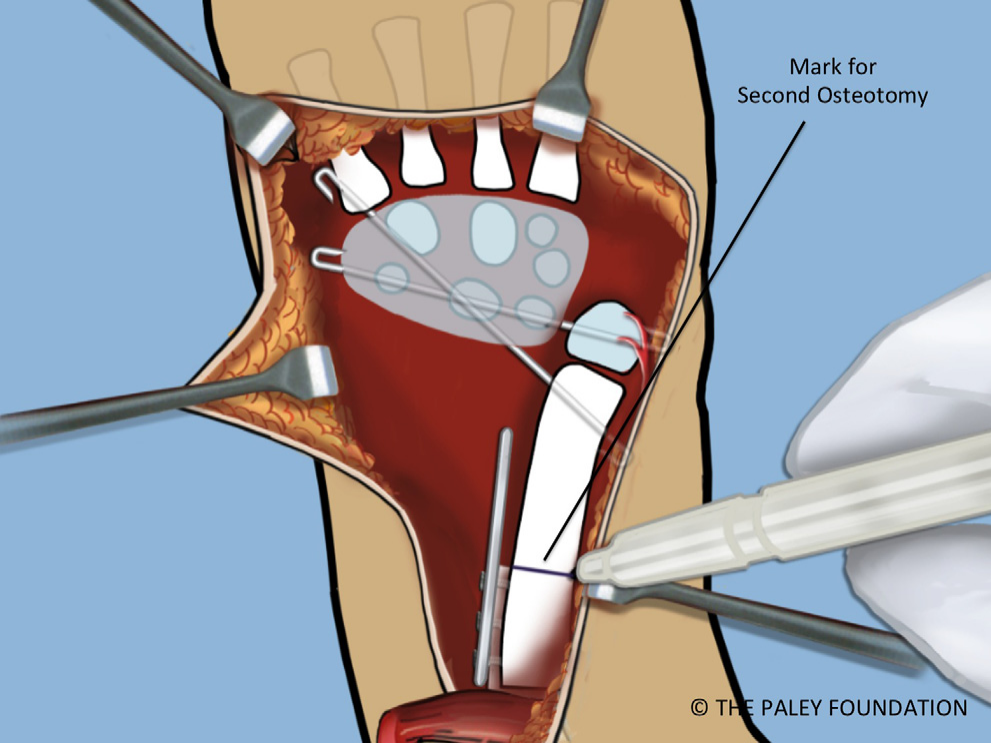
With the bone ends overlapped and the wrist pinned, the level of the shortening osteotomy should be marked.

**Figure 20.**
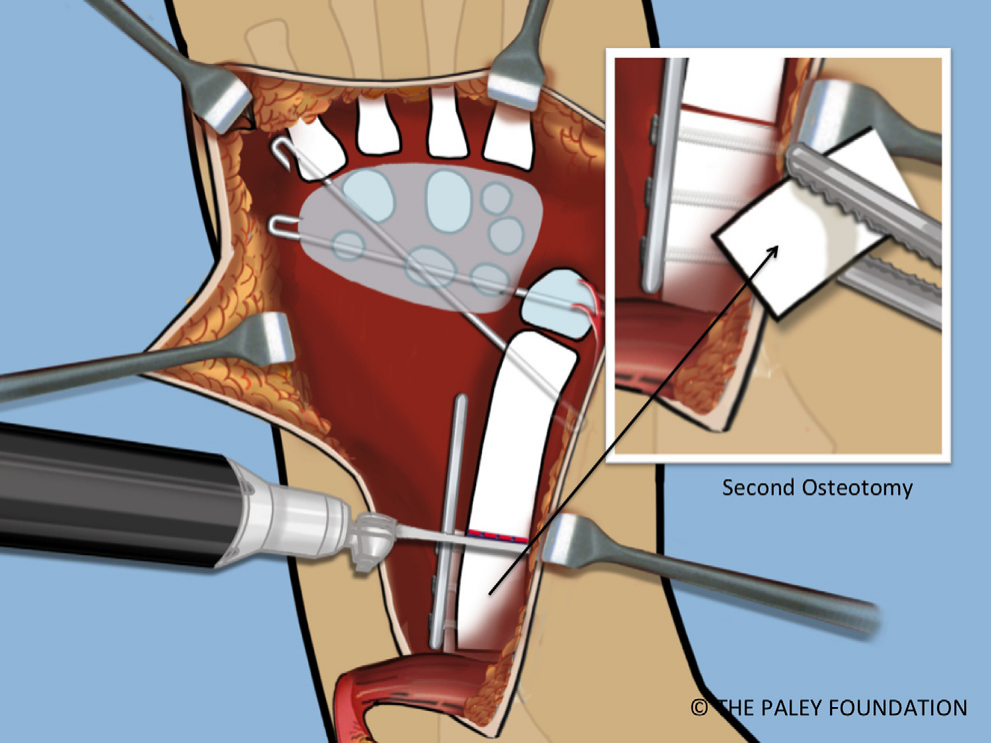
The shortening osteotomy is performed with a saw.

Step 20: Mark the amount of overlap of the bone ends. This is the amount that needs to be shortened. Make a second osteotomy of the ulna at this mark (Figures 19 and 20).

**Figure 21.**
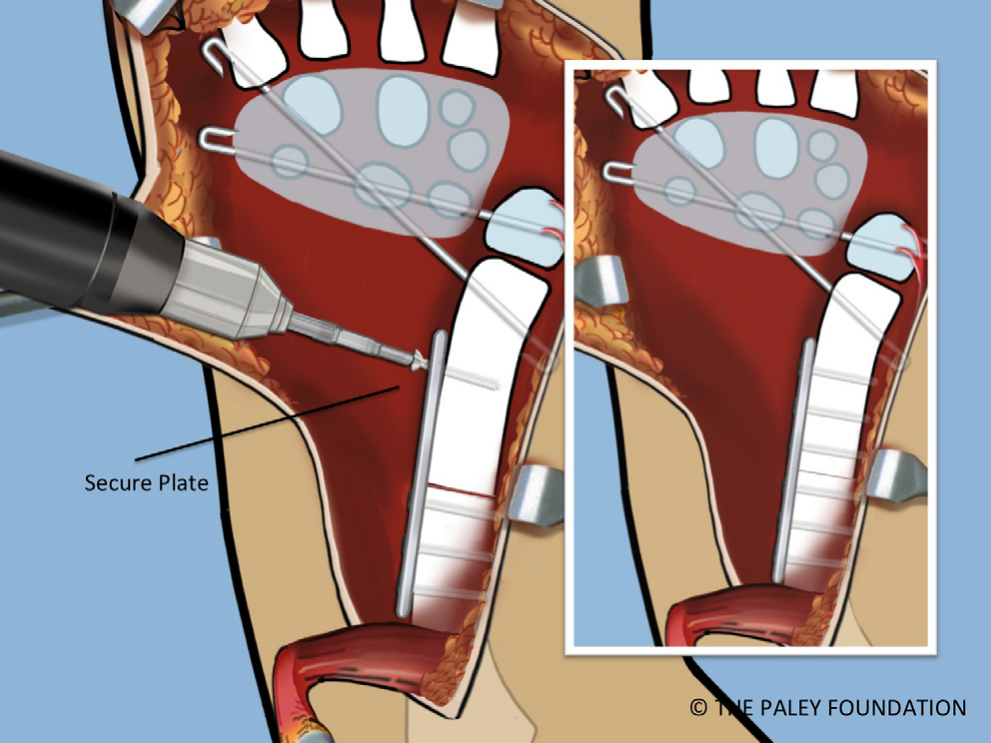
Fix the distal ulna to the plate. Adjust the rotation so that the plane of the hand and the plane of the elbow motion are the same. Stated differently, the lateral the profile view is a lateral of the elbow with an AP of the hand.

Step 21: Adjust the rotation of the ulna so that the hand is in the plane of flexion and extension of the elbow. Fix the ulna it to the plate in this position with two or three screws (Figure 21).

**Figure 22.**
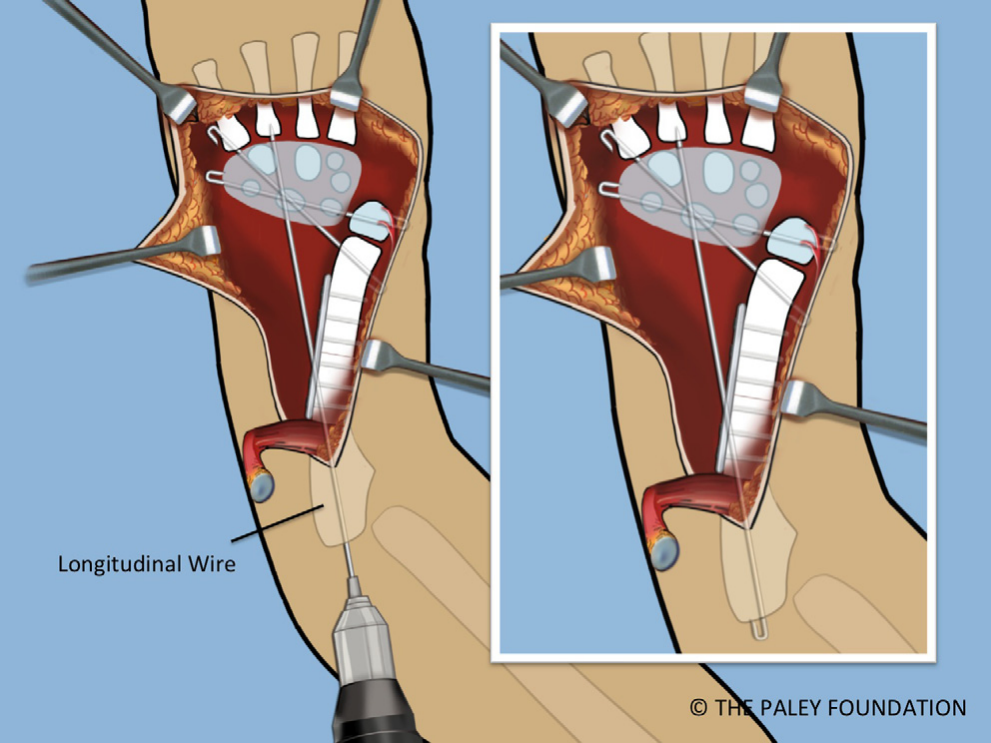
A third *temporary wrist arthrodesis* wire is inserted from the olecranon in a longitudinal antegrade fashion to exit the ulna and transfix the carpus. This wire is also cut and curled proximally.

Step 22: Insert an antegrade 1.5 wire into the olecranon and advance it distally until it comes out the midulna through the dorsal cortex. Advance this wire until it enters the carpus and stabilizes it. The proximal end of this wire should be curled over, cut and buried at the olecranon. The three wires holding the wrist are called temporary arthrodesis wires (Figure 22).

**Figure 23.**
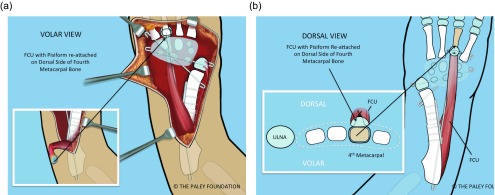
The FCU should be transferred to the dorsum of the hand. This is shown from the volar (a) and dorsal (b) and cross-sectional views (b). The pisiform bone is sutured to the base of the 4th metacarpal. A non-absorbable suture is passed around the base of this metacarpal.

Step 23: Dissect the dorsum of the hand free of its soft tissues at the level of the metacarpals. Dissect a space around the dorsal base of the 4th metacarpal and roughen up the surface down to bone. Also expose the volar aspect of this metacarpal (Figure 23).

Step 24: Cut off the volar articular cartilage of the pisiform with a knife. Pass the pisiform deep to the ECU and EDM and the dorsal cutaneous branch of the ulnar nerve. Pass a 2-0 non-absorbable suture from dorsal to volar through the pisiform. Pass the needle through the interspace between the 3rd and 4th metacarpal bases from dorsal to volar. Re-pass the same needle from volar to dorsal through the interspace of the 4th and 5th metacarpal bases. Pass the needle from the cut volar surface of the pisiform through to the dorsal surface. Make sure that the loop of the suture does not entrap any soft tissues as it is pulled down to bone. Pull the pisiform down to the dorsal base of the 4th metatarsal (Figure 23).

**Figure 24.**
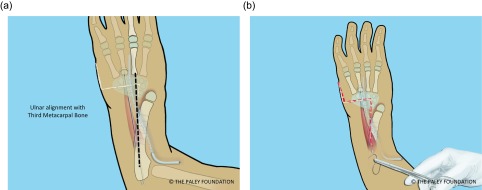
The proximal ulna is parallel and in line with the third metacarpal (a). The Z-shaped incision is closed over a drain (b). It is important to complete a volar fasciotomy before closing to allow for swelling without the risk of compartment syndrome.

Step 25: Perform a volar fasciotomy of the forearm up to the elbow (Figure 24).

Step 26: Remove the tourniquet. Close the wound over a drain (Figure 24).

Step 27: An occupational therapy ulnar gutter orthoplast splint is custom molded in the operating room and secured over the sterile dressing with Velcro straps. These straps can be adjusted to allow for the expected swelling that occurs. The splint should support the fingers in near full extension.

#### Postoperative management

The arm is strictly elevated, circulatory checks and splitting of any circumferential dressing are carried out. Pain control is accomplished with intravenously administered narcotics and then switched to oral medication. Physical therapy begins on postoperative day one and consists of elbow and finger range of motion (ROM) exercises. After six weeks replace the long arm splint with a below elbow ulnar gutter splint. The temporary arthrodesis wires of the wrist should be removed after three months. This can be combined with pollicization of the index finger surgery when needed. The ROM of the wrist both active and passive can begin at this time. The therapy to train the wrist for dorsiflexion using the FCU should commence after the removal of these wires.

## Results

In 2008, Paley et al. reported on 21 hands in 14 consecutive patients who underwent ulnarization between 2000 and 2006 [19]. The age of surgery ranged from one to 14 years (mean 6 years.). The average follow-up was 46 months (15–91 months). No patient had any recurrence of deformity or necrosis/growth arrest of the ulnar epiphysis. Wrist dorsiflexion (passive) improved from an average of −15° to 36°. The elbow ROM remained unchanged, but the arc of motion and flexion contracture increased postoperatively. The hand-to-forearm angle improved from an average of 53° of radial deviation to 22° of ulnar deviation. The hand-to-forearm position improved from an average of 16 mm of radial displacement to 21 mm of ulnar displacement. The palmar carpus displacement improved from an average of 8 mm volar to the ulna to 6 mm dorsal to the ulna. The ulnar length improved from an average of 79 mm to 102 mm during the follow-up period. There were two incision complications that required debridement and/or secondary wound closure and there were five external fixation pin infections that required an antibiotic treatment. Parents of all children stated that the cosmetic appearance and function of the hand improved after surgery.

In a subsequent clinical follow-up of many of these original ulnarization patients, Paley has observed that 10–15% developed an ulnar deviation drift due to gravity. This went away when the hand was used due to muscle pull. This may be related to the unsupported position of the carpus since the head of the ulna is now on the radial side. The other late observation on the children who reached adolescence is that the prominence of the head of the ulna became more pronounced. This is related to unimpeded active growth of the head of the ulna relative to the more tethered carpus and hand. In some children, this was managed by the resection of prominent bump at the end of growth.

**Figure 25.**
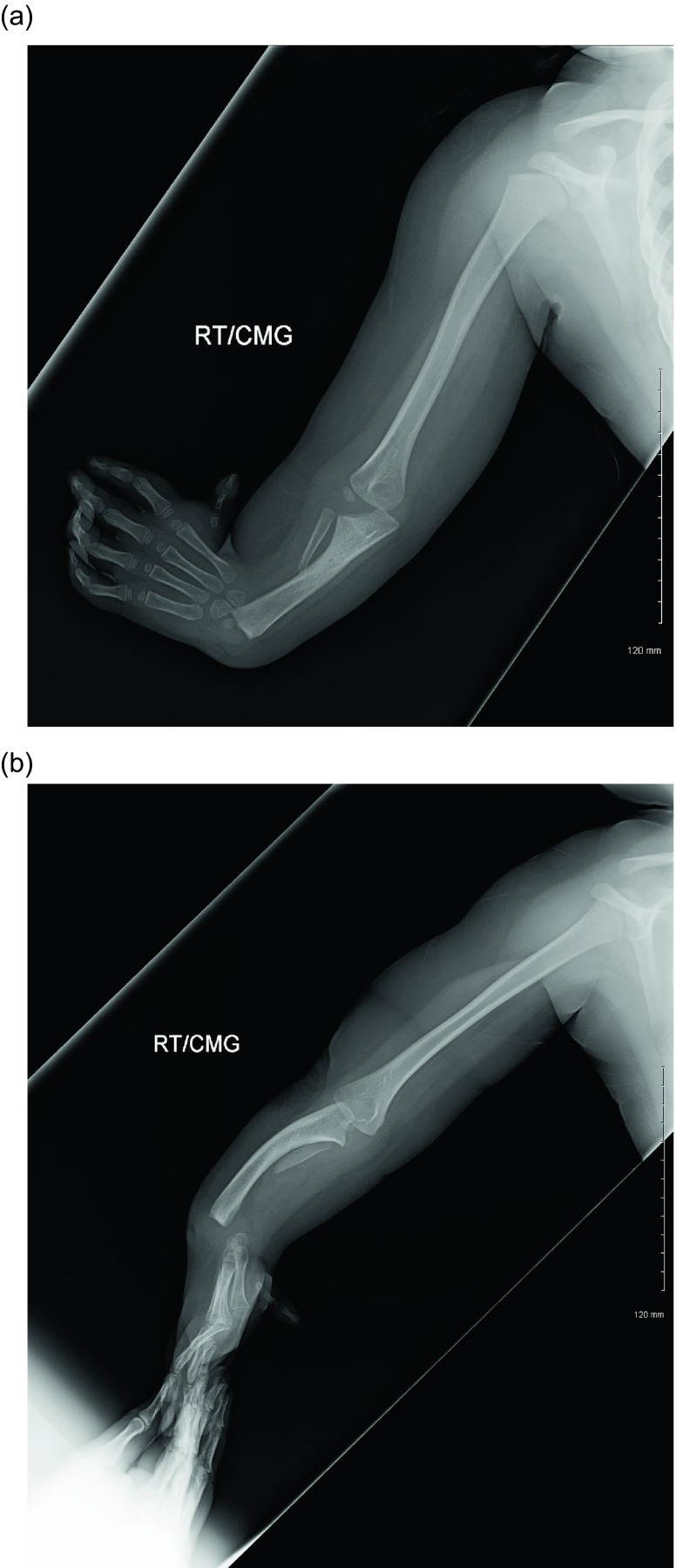
AP and lateral radiographs of left hand of a four-year-old boy with radial club hand before surgery.

**Figure 26.**
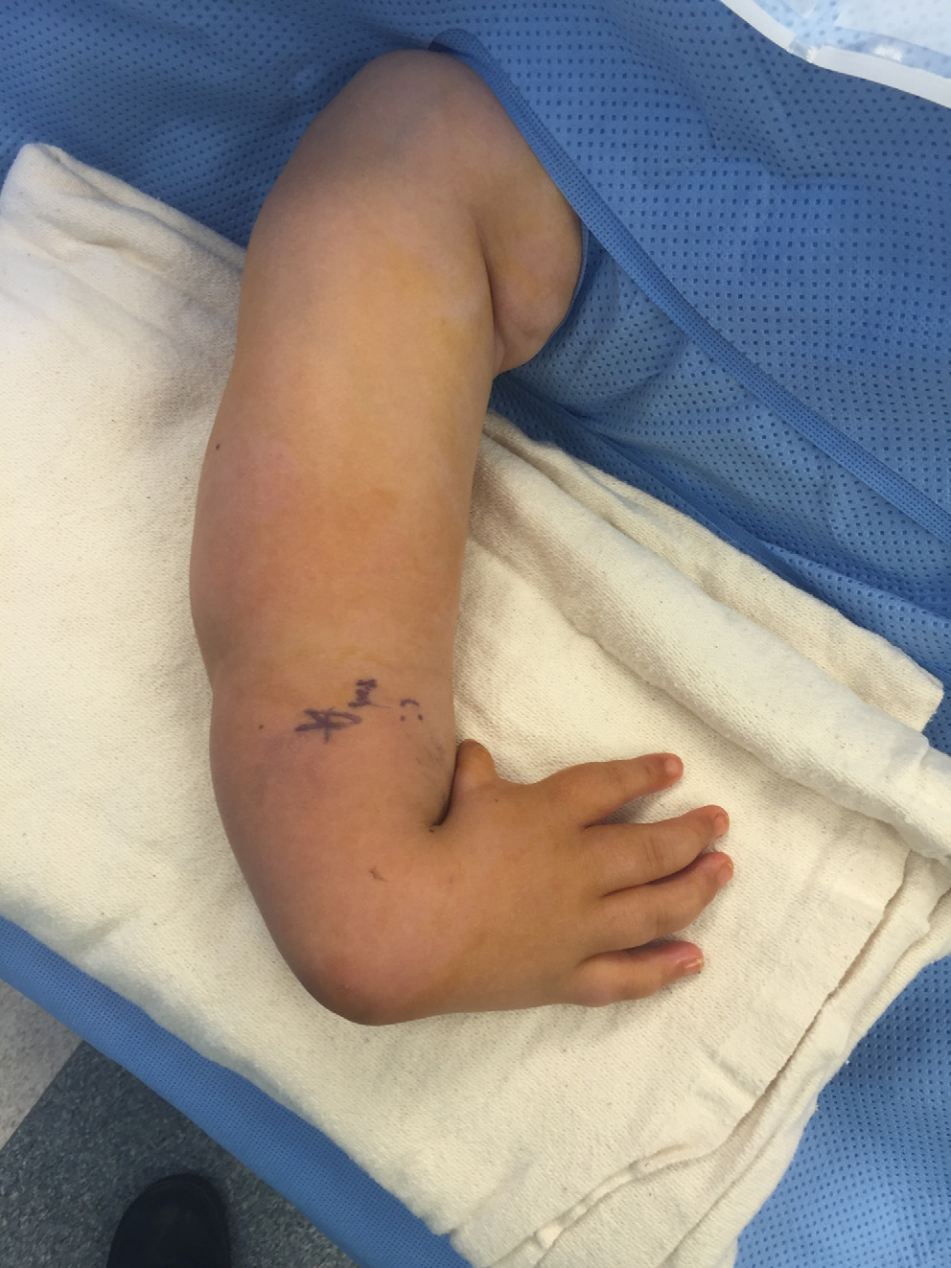
Photograph of the hand before surgery.

**Figure 27.**
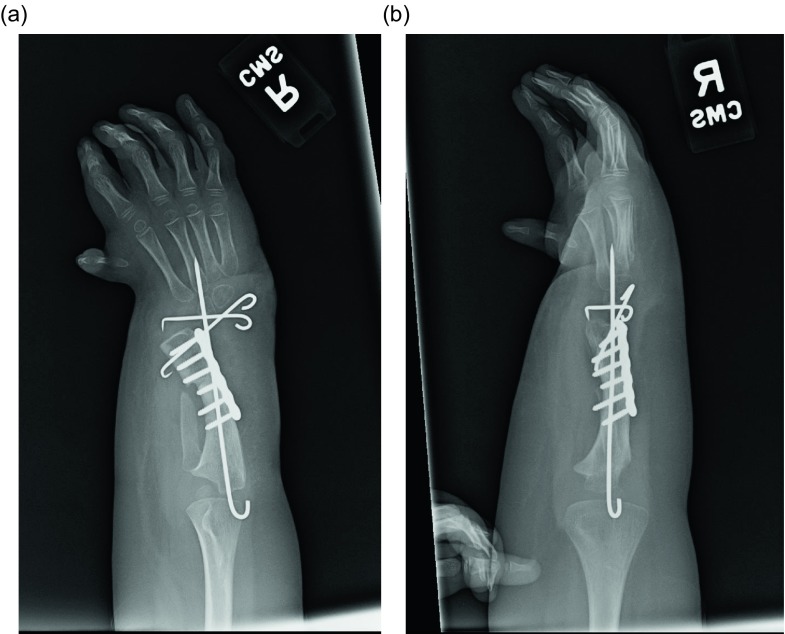
AP and lateral radiographs after ulnarization with shortening. Note the temporary arthrodesis wires in place and the plate on the ulna.

**Figure 28.**
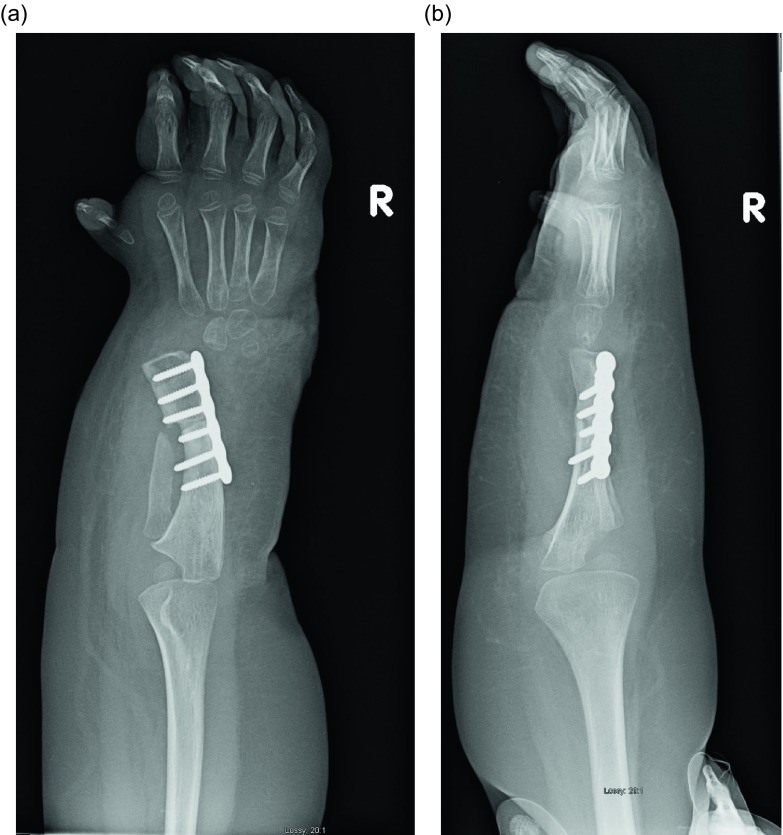
AP and lateral radiographs after removal of the wires. Note that the ulnar head remains at station.

**Figure 29.**
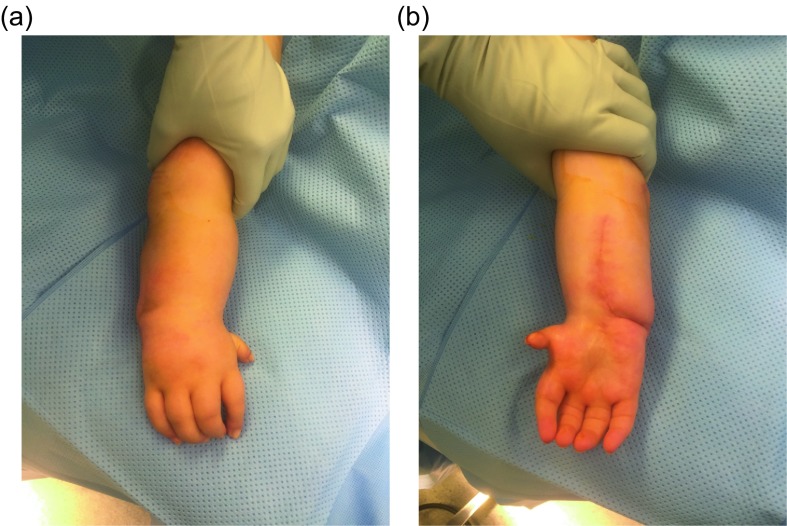
Final photographs, dorsal view (a), volar view (b), showing the hand after ulnarization with shortening.

The new ulnarization procedure, which placed the carpus more on top of the side of the head of the ulna rather than completely on the side of the head of the ulna, protects from the overgrowth of the ulna (Figures 25–29). This preliminary report is too short a follow-up (two years) and cannot yet evaluate this. The original ulnarization study of Paley et al. [19], with its average four-year follow-up results, failed to identify the overgrowth of the ulnar head relative to the hand. Only a long-term follow-up of skeletal maturity can document such findings.

## Discussion

In 1893, Sayre [20] placed a sharpened distal end of the ulna into a notch created in the carpus by excising the lunate and the capitate. Many authors reported variations of this technique of centralizing the carpus on the end of the ulna [1, 3–5, 7, 9, 10, 20, 21]. The techniques include release of the tight tethering radial soft tissues, removal and notching of carpal bones, circumferential dissection and paring down of the head of the ulna, pinning of the carpus to the head of the ulna and tendon transfers.

Most authors have agreed that the appearance of the deformed upper extremity is improved after centralization [1, 4]. Improvement of specific functional activities after centralization is difficult to document for a growing child because children generally adapt well to the limitations of congenital deformities. Even though good early results have been reported, late recurrence of deformity and revision remains a common problem. Watson et al. [5] reported a follow-up ranging from one to 20 years, with recurrent deformity of radial deviation of 15° to 30° in 7 of 12 limbs. Bora et al. [7] reported the results of 14 limbs (eight patients) treated by centralization without arthrodesis at an average follow-up period of 14.6 years. They accepted a persistent mean radial angulation of 35° and concluded that the centralization procedure was satisfactory. Lamb [4] reported on the centralization of 31 upper limbs with an average of five-year follow-up. They reported a persistent average radial angulation of 22° at the final follow-up visit and three cases of recurrent volar flexion deformity. Bayne and Klug [22] reported six revision centralizations in 53 centralization procedures with a minimum follow-up period of 36 months; their final results included 10 limbs with poor outcomes and 43 with good or satisfactory outcomes. Manske et al. [9] presented a report on the 21 centralization procedures with an average follow-up period of 25 months. They noted two revisions and six limbs with a radial angulation of more than 35°. Damore et al. [14] presented a report of 19 centralization procedures with an average follow-up period of 6.5 years. They reported four revisions and recurrence of radial angulation in most of the cases. Geck et al. [15] reported the results of 15 centralization and 14 modified radialization procedures at an average follow-up period of 50 months. They reported a 33% recurrence revision rate at five years. Other ubiquitous complications of centralization include growth arrest of the distal ulna, ankylosis of the wrist, and recurrent instability of the wrist [1].

Buck-Gramcko [11, 13] reported that there was a high recurrence and growth arrest rate in his 63 centralizations performed between 1969 and 1979. In 1979, he modified the procedure to what he later published and named *radialization*. With radialization the distal ulna was positioned radial to the carpal bones and not at its center. He postulated two advantages with the radialization procedure: preserving the carpal bones maximized wrist function; deformity recurrence was a result of muscle imbalance resulting from the short and contracted radial wrist muscles (flexors) and a long radial lever arm. He recommended transferring the flexor and extensor carpi radialis tendons ulnarly to increase the ulnar lever arm. Buck-Gramcko reported a 7.5% recurrence and many spontaneous arthrodeses of the wrist as well as numerous wound complications. He also reported 11% avascular necrosis and growth arrest of the distal ulnar epiphysis [13]. Geck et al. [15] compared the results of centralization (14 limbs) and radialization (15 limbs) at an average follow-up period of 50 months. Six of 14 limbs that underwent centralization required revision. Two of 15 limbs that underwent radialization required revision. Although they did not find statistical significance between the groups, they concluded that the data support the hypothesis that a more ulnar translation and ulnar angulation of the wrist reduce the radial lever arm and thus the incidence of recurrence.

The ulnarization procedure is based on the original radialization concept presented by Buck-Gramcko [11]. The term *ulnarization* refers to the direction of displacement of the carpus relative to the ulna. Several important differences exist between ulnarization and radialization. Ulnarization is performed through a volar approach to the wrist while radialization is described through a dorsal approach. The volar approach allows excellent visualization of all the critical neurovascular structures for the procedure to be performed safely. The median, ulnar and radial neurovascular bundles, dorsal cutaneous branch of the ulnar nerve, FCU tendon, and pisiform are easily identified. The contracted soft-tissue structures are on the radial and volar side of the radial clubhand. We can identify and resect the radial anlage, if present, and release the short fibrotic radial muscles and fascia. The complete radial and volar soft-tissue dissection and release facilitates translocation of the carpus to the ulnar side. The caput ulna vessels can also be protected avoiding injury to the circulation of the distal ulna. The second major and original difference of ulnarization from radialization is the transfer of the FCU tendon with the pisiform to the dorsal aspect of the hand. Paley first described using the FCU tendon transfer for radial club hand (RCH) in 1998, in a book chapter in a congenital hand deformity book edited by Buck-Gramcko [23]. This transposition eliminates the FCU as a deforming force and converts it into a corrective balancing force. Dorsiflexion is weak in RCH and by transferring the FCU, it serves as a strong dorsiflexor. Buck-Gramcko recommends transferring the FCR. In the author’s experience, the FCR is usually absent but the FCU is always present and well developed. The RCH children have weak grip strength caused by the volar posture of the hand. The strong and reliable FCU tendon transfer splints the hand in a slight dorsiflexed posture to allow better flexion of the fingers during grip. This improves grip strength.

The Paley et al. [19] study of the original ulnarization without shortening had no recurrences of radial deviation and no growth arrest of the distal ulna. The follow-up of these patients to the current date has maintained these findings. If the recurrence of radial deviation deformity and growth arrest are considered the benchmarks of success of the procedure, ulnarization with or without shortening produces the best results when compared to radialization and centralization.

Distraction has been used as an adjunct to centralization and radialization with significant decrease in growth arrest of the distal ulna but with persistent high incidence of recurrent radial deviation. The use of an axial pin to prevent recurrent radial tilt does prevent recurrence but also does not permit wrist motion [24]. As an alternative to centralization or radialization the Vilkki procedure developed in 1992 [25] performs a microvascular transplant of the second toe and metatarsal to support the radial side of the wrist. It too is a two-stage procedure that employs distraction as the first-stage surgery followed by the microvascular toe transfer. The Vilkki procedure results in persistent radial deviation of an average of 28° and recurrent radial deviation of 12° in the long term [26]. Growth of the distal ulna was well preserved and the wrist range of motion was excellent. The Vilkki procedure requires sacrifice of the second ray and toe and is a much more invasive and risk-associated procedure than ulnarization. Ulnarization is a much simpler, lower risk procedure that can be performed by most hand surgeons without microvascular expertise.

## Conclusions

With greater follow-up, since the author’s original study was performed, the biggest problems observed with ulnarization have been overgrowth of the distal ulna relative to the carpus and excessive ulnar deviation. To avoid this, the distraction to place the carpus more distally on the head of the ulna should be avoided. Instead, the ulna should be shortened. This automatically places the head of the ulna adjacent to the side of the scaphoid. This avoids a prominent head of the ulna and also avoids relative overgrowth. There also seems to be less overcorrection with this method. Thus far, the newer ulnarization method shows no evidence of recurrence or growth arrest but also no evidence of ulnar deviation deformity or a prominent radial bump. Ulnarization with distraction has already stood the test of time. Ulnarization without distraction but with diaphyseal shortening is a promising improvement in the original technique.

## Conflict of interest

The author declares no conflict of interest in relation with this paper.
